# Modeling the impact of changes in day-care contact patterns on the dynamics of varicella transmission in France between 1991 and 2015

**DOI:** 10.1371/journal.pcbi.1006334

**Published:** 2018-08-01

**Authors:** Valentina Marziano, Piero Poletti, Guillaume Béraud, Pierre-Yves Boëlle, Stefano Merler, Vittoria Colizza

**Affiliations:** 1 Center for Information Technology, Bruno Kessler Foundation, Trento, Italy; 2 Médecine Interne et Maladies Infectieuses, Centre Hospitalier de Poitiers, Poitiers, France; 3 EA2694, Université Droit et Santé Lille 2, Lille, France; 4 Interuniversity Institute for Biostatistics and statistical Bioinformatics, Hasselt University, Hasselt, Belgium; 5 INSERM, Sorbonne Université, Institut Pierre Louis d’Epidémiologie et de Santé Publique IPLESP, Paris, France; The Pennsylvania State University, UNITED STATES

## Abstract

Annual incidence rates of varicella infection in the general population in France have been rather stable since 1991 when clinical surveillance started. Rates however show a statistically significant increase over time in children aged 0–3 years, and a decline in older individuals. A significant increase in day-care enrolment and structures’ capacity in France was also observed in the last decade. In this work we investigate the potential interplay between an increase of contacts of young children possibly caused by earlier socialization in the community and varicella transmission dynamics. To this aim, we develop an age-structured mathematical model, informed with historical demographic data and contact matrix estimates in the country, accounting for longitudinal linear increase of early childhood contacts. While the reported overall varicella incidence is well reproduced independently of mixing variations, age-specific empirical trends are better captured by accounting for an increase in contacts among pre-school children in the last decades. We found that the varicella data are consistent with a 30% increase in the number of contacts at day-care facilities, which would imply a 50% growth in the contribution of 0-3y old children to overall yearly infections in 1991–2015. Our findings suggest that an earlier exposure to pathogens due to changes in day-care contact patterns, represents a plausible explanation for the epidemiological patterns observed in France. Obtained results suggest that considering temporal changes in social factors in addition to demographic ones is critical to correctly interpret varicella transmission dynamics.

## Author summary

During the last decades, an increasing circulation of varicella in the early childhood has been observed in France. A plausible explanation of this trend may rely on the progressive increase of day-care attendance in the past years, which could have anticipated the exposure of young children to the infection. We propose a retrospective modelling study to assess whether the varicella dynamics in France since 1991 can be explained in terms of increasing day-care contacts of children under 3 years of age. To this aim, we develop a model including demographic changes and variations in age-specific contact rates over time. Our findings suggest that a 30% increase of day-care contacts in early childhood can explain the observed epidemiological trends. Obtained results highlight that temporal changes in contact patterns can significantly affect the transmission of childhood infectious diseases and should therefore be considered when investigating medium and long-term epidemiological patterns. A better understanding of the interplay between changing social behavior and disease transmission can help the interpretation of surveillance data and the design of effective and targeted intervention strategies.

## Introduction

Varicella is a vaccine-preventable infectious disease caused by exposure to Varicella-Zoster Virus (VZV). The pathogen is antigenically stable so that, in principle, no changes in transmission or immunogenicity caused by mutations of the virus are expected over time [1]. In France, about 90% of the population gets infected with varicella before 8 years of age; most of infections occur in the early childhood and result in relatively mild symptoms [2,3]. In this country, vaccination against varicella is not recommended and little used in children. Previous studies have shown that temporal changes in the crude birth rate of a population are key drivers of the dynamics of childhood infectious diseases, such as varicella and measles, by affecting the replenishment of susceptible individuals in the population [4–8]. Since the early 90s, France experienced a roughly constant crude birth rate [9] after a strong demographic transition in the last century characterized by a progressive decline of birth and death rates. This corresponded to stable varicella infection rates at the population level between 1991 and 2015, as revealed by the French GPs Sentinelles Network for surveillance [10]. However, when looking at the distribution of cases by age, surveillance data highlight that during this period varicella incidence has increased in children aged 0–3 years and decreased in children aged 4–7 years. A similar pattern has been detected in other countries, including Slovenia, the US and England [11–16], where varicella incidence doubled in children aged 0–4 years between 1983 and 1998 and halved in those aged 5–14 years. This suggests that, beyond changes in fertility and mortality rates, other factors may influence the circulation of childhood infections across the different age segments of the population. One of them is variations in the population mixing patterns driven by socio-demographic changes, affecting school attendance and household structure. In particular, some epidemiological studies have hypothesized that an increase of varicella incidence in young ages may be ascribable to increased social contacts in these age groups, possibly caused by earlier inclusion in nurseries or day-care centers [11–13]. Past modeling efforts, based on a theoretical framework assuming a stationary age distribution of the population, have suggested that a substantial increase of contact rates in preschool children is consistent with the increase in varicella consultations in UK observed between 1970 and 1998 in this age segment [17].

The aim of this work is to assess whether changes in the age-specific varicella incidence observed in France can be the result of an increase of contacts in the early childhood.

To this aim, we considered two transmission models with the same demographic and epidemiological structure, but differing in mixing patterns over time. Both models take explicitly into account demographic changes occurred during the last century [9]. In the first model, mixing patterns between individuals of different ages are assumed to remain constant over time and are modelled according to the age-specific contact matrix estimated for France in 2012 [18]. In the second model, we assume a linear increase of contact rates occurring at day-care facilities for children under 3 years of age, starting in the decades before 2012.

## Methods

### Demographic and epidemiological model

The adopted modelling approach is based on a deterministic age-structured model similar to the one developed by previously published studies to investigate historical dynamics of measles across different countries and varicella in Spain [5–7]. The population, grouped into 1-year age classes (0–89+), is initialized in 1850 at the demographic and epidemiological equilibrium. The latter was obtained by running the transmission model with constant crude birth and mortality rates fixed to those observed in 1850, and by initializing the system with 10 infected individuals in a fully susceptible population. Simulations of varicella dynamics from 1850 to 2015 are obtained by running the model as informed by the yearly variations of birth and age-specific mortality rates provided by the National Institute of Statistics and Economical Studies (INSEE).

The demographic model is validated against the age distribution of the population observed in France during the simulated period (1850–2015) [9]. Realistic mixing patterns by age are modelled using contact matrices estimated for France in 2012 [18]. Age-specific contact matrices are here defined as the average number of unique physical and conversational contacts with individuals of different ages occurring daily, regardless their duration and frequency [18].

The transmission of varicella follows an MSIR model. Briefly, maternal antibodies protect new-borns against varicella infection (M) for 2 months on average [19], after which they become susceptible to varicella infection (S). Susceptible individuals are exposed to a time- and age-dependent force of infection *λ*_*i*_(*t*) as follows:

$$\lambda_{i}(t)=\beta \sum_{j=0}^{n}C_{ij}\left(t\right)\frac{I_{j}(t)}{N_{j}(t)}$$ (1)

where *t* and *i* denote time and the individuals’ age, respectively; *n* = 89 years is the maximum age considered in the model, *I*_*j*_(*t*)/*N*_*j*_(*t*) is the fraction of individuals of age *j* who are infected at time *t* and *C*_*ij*_(*t*) is the contact matrix at time *t*, which is defined as the average number of contacts of an individual of age *i* with individuals of age *j*; finally, under the social contact hypothesis [20], *β* represents an age-independent constant proportionality factor driving the contribution of individuals’ contacts to the transmission of the infection.

Once recovered, varicella infected individuals (I) acquire life-long immunity against varicella. The generation time of varicella is assumed equal to 3 weeks on average [21].

Epidemiological and demographic transitions occurring within a given year are described by the following set of ordinary differential equations:

$${\dot{M}}_{i}(t)=\delta_{i0}b(t)N(t)-\omega M_{i}(t)-\mu_{i}(t)M_{i}(t)$$

$${\dot{S}}_{i}(t)=\omega M_{i}(t)-\lambda_{i}(t)S_{i}(t)-\mu_{i}(t)S_{i}(t)$$ (2)

$${\dot{I}}_{i}(t)=\lambda_{i}(t)S_{i}(t)-\gamma I_{i}(t)-\mu_{i}(t)I_{i}(t)$$

$${\dot{R}}_{i}(t)=\gamma I_{i}(t)-\mu_{i}(t)R_{i}(t)$$

where *b*(*t*) is the yearly crude birth rate, *δ*_*ij*_ is the Dirac delta function, *N*(*t*) is the population size, *ω* is the waning rate of maternal antibodies, *μ*_*i*_(*t*) is the yearly age-specific mortality rate and *γ* is the recovery rate from varicella infection. At the end of each year, the age of individuals is incremented by 1.

### Contact matrices over time

In this work, we consider two variations of the model described in the previous section, which differ in the assumption made to model mixing patterns during the past.

In model M1, at each time *t* the average number of contacts of an individual of age *i* with individuals of age *j* is computed as:

$$C_{ij}(t)={\bar{C}}_{ij}^{S}+{\bar{C}}_{ij}^{O}$$ (3)

Where ${\bar{C}}_{ij}^{S}$ and ${\bar{C}}_{ij}^{O}$ are respectively the matrices of contacts within and outside schools estimated for France in 2012 [18]. School contacts of individuals younger than 3 years of age correspond to social interactions occurring at pre-school facilities, such as day-care centers.

In this sense, model M1 assumes no changes in mixing patterns of individuals over time.

In Model M2, we account for potential temporal changes in contact patterns by adding a time-dependent scaling factor to the matrix of school contacts. Specifically, we assume that $C_{ij}(t)=f_{ij}(t){\bar{C}}_{ij}^{S}+{\bar{C}}_{ij}^{O}$ where

$$f_{ij}(t):=\left\{ \begin{array}{c} 1-\alpha (2012-t)\;if\;i<4\;or\;j<4\\ 1\;elsewhere \end{array}\right.$$ (4)

This simple assumption accounts for linear temporal changes of contact rates in children below 3 years of age and aims at illustrating the potential impact of an increased attendance at day-care centers.

By keeping track of the contribution of different age segments and different settings to the age specific force of infection, we disentangle the proportion of varicella infections caused by school contacts (at different school levels, including day care) and compute the infection matrices representing the proportion of varicella cases generated by contacts of susceptible individuals of age *i* with infected individuals of age *j*.

### Model estimates

Free parameters of the two models were estimated separately through a Markov chain Monte Carlo (MCMC) approach applied to the negative binomial likelihood of the yearly age-specific incidence of varicella observed in France over the period 1991–2015 [10]. The two models have the following free parameters in common: the transmission scale factor (*β*), the varicella reporting rate, which is assumed constant over age and time, and the over-dispersion of the negative binomial distribution. Model M2 has an additional free parameter shaping the changes in school contacts of young children (*α*). Models’ performances were compared using the Deviance Information Criterion (DIC) and the Akaike Information Criterion (AIC). Further details on model formulation and estimation are provided in S1 Text.

## Results

### Data analysis

The analysis of observed varicella incidence by age group reveals a statistically significant increase of infection rates in children less than 3 years old and a significant decrease in those older than 5 years (see S1 Text). Temporal changes in the infection rates in new-borns, in children of 4 years of age and in total yearly incidence were found to be not statistically significant.

### Model estimates

According to both measures used to assess model performances, model M2 (DIC: 6508.8; AIC: 6513.5) is able to better represent the data than model M1 (DIC: 6513.4; AIC: 6523.8).

**Fig 1:**
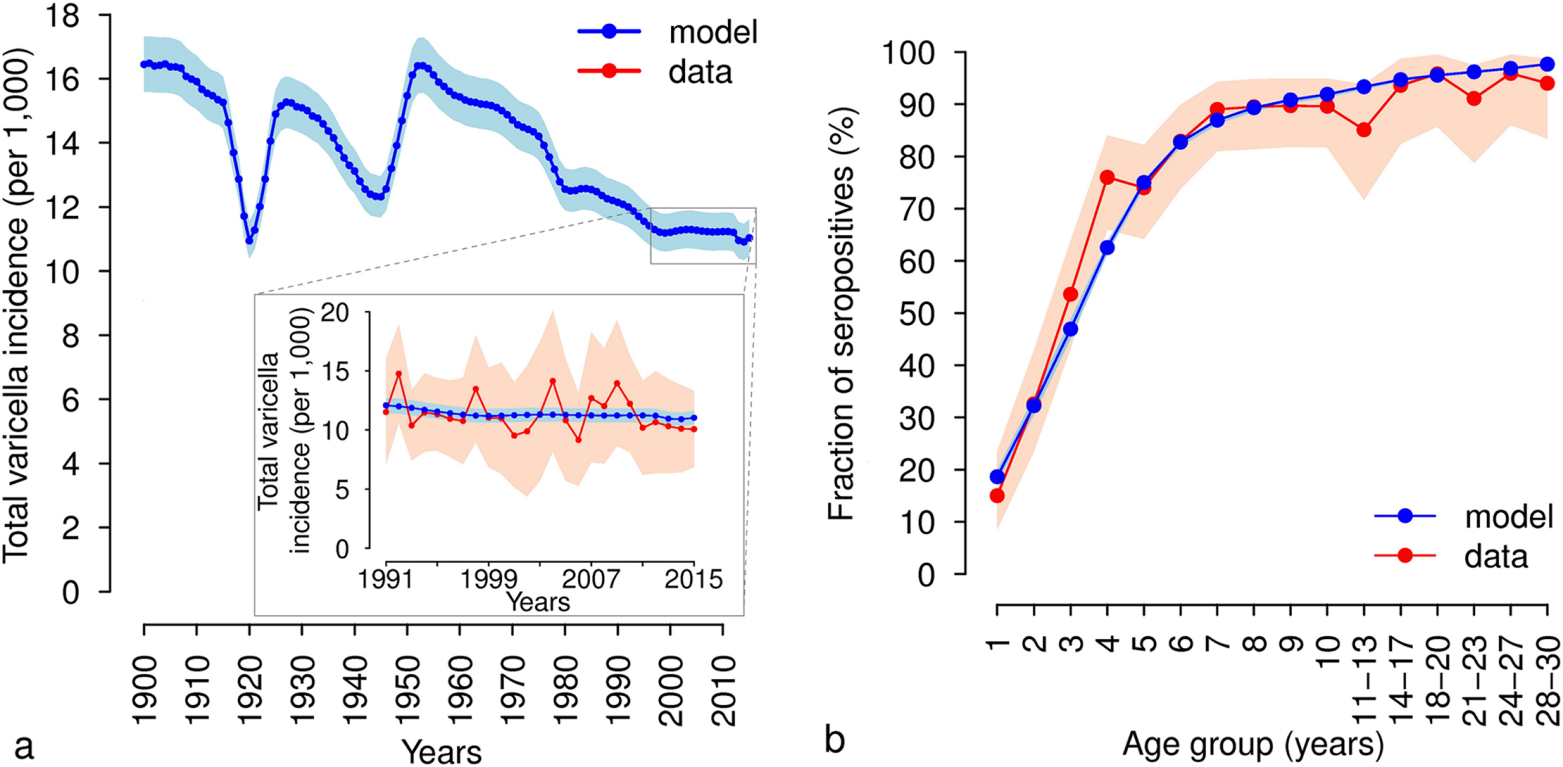
Epidemiological validation of Model M2. **a** Mean total incidence of varicella (per 1,000 individuals) as estimated by model M2 over the period 1900–2015. Shaded areas represent 95% CI of model estimates. The inset compares the total incidence of varicella predicted by the model in 1991–2015 (blue) to the one observed by the French GPs Sentinelles Network over the same period (red) [10]. Shaded areas represent 95% CI of model estimates (light blue) and of the data (orange). **b** Age-specific VZV seroprevalence as observed in data from France in 2003 [2] (red) and as estimated by model M2 (blue). Shaded areas represent 95% CI of the data as computed by exact binomial test in [2] (orange) and 95% CI of model estimates (light blue).

Both models considered in our analysis are able to reproduce changes in the overall size and age distribution of the French population as observed during the last century (details shown in S1 Text). Briefly, in agreement with available demographic records, the simulated population dynamics shows that the progressive decrease in the crude birth rate experienced between 1900 and 2015 led to a significant reduction of the fraction of children in the population. Consistently with previous works [5,6] and in agreement with observations [10], estimates from both models suggest that the total incidence of varicella did not significantly change between 1991 and 2015, when the yearly crude birth rate remained approximately stable (model M2, Fig 1A; model M1 shown in S1 Text). The estimated varicella reporting rate ranges between 90.7% (95% CI: 85.8%-96.0%) in model M1 and 88.6% (95% CI: 84.1%-93.1%) in model M2. The simulated varicella dynamics were validated against an independent dataset, represented by the age-specific VZV serological profile observed in France in 2003 [2]. According to our simulations and consistently with data, in 2003 about 30% of 2-year-old children were immune to varicella and this fraction increases up to 87% by age 7 (model M2, Fig 1B; model M1, in S1 Text). These results suggest that the overall varicella circulation observed in France during the last decades can be explained in terms of the relatively stable crude birth and mortality rates characterizing this period and does not depend on possible changes in mixing patterns.

**Fig 2:**
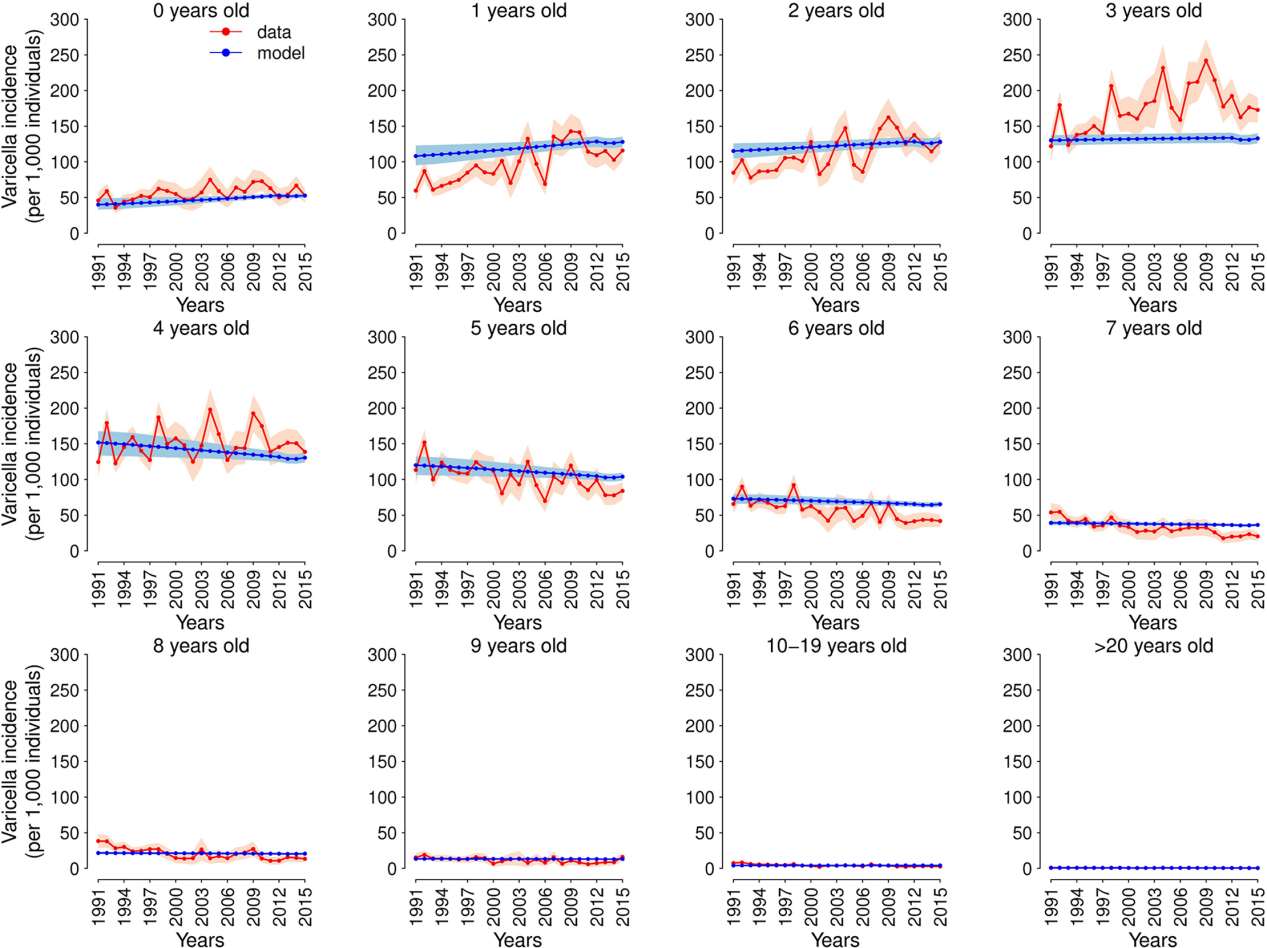
Incidence of varicella by age (1991–2015). Incidence of varicella by age as observed in France between 1991 and 2015 (red) and as estimated by model M2 (blue). Shaded areas show the 95% CI of data and of model estimates.

However, trends in the age-specific varicella incidence estimated with the two models are remarkably different. A detailed analysis on the ability of models M1 and M2 in reproducing the observed dynamics is reported in S1 Text. In particular, model M1 that does not account for changes in mixing patterns over time yields stable infection rates in all age groups between 1991 and 2015 (see S1 Text). This model formulation thus fails to capture the changes in the age distribution of varicella cases observed in the period under study. In contrast, model M2, by explicitly taking into account possible changes in the rate of contacts of young children (0–3 years) established in day-care structures in the years prior to 2012, reproduces the observed trends in the age-specific incidence of varicella (see Fig 2). In particular, model M2 estimates a 12.1% increase of varicella incidence in 0–3 years old children over 1991–2015 and a 13% decrease of varicella incidence in older age groups over the same period. The estimated increase in varicella incidence among 0–3 years is the result of a 18% increase in 1-year old children, 11% in 2-year old children, and only 2% in 3-year old children (Fig 2, first row). The estimated changes of varicella transmission dynamics are ascribable to an increase in the average number of day-care contacts during the last decades. According to our estimates, in 1991, day-care contacts represented on average the 15.5% of the total contacts of children aged 0–3 years, while this fraction increased up to 19.2% in 2012 (details are provided in S1 Text).

**Fig 3:**
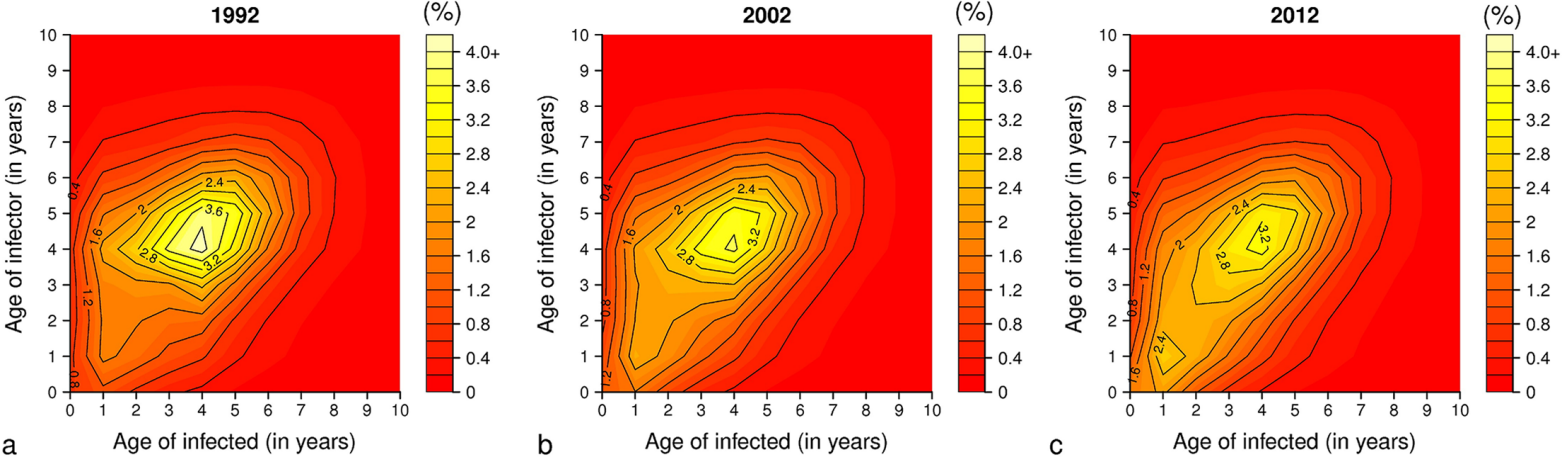
Infection matrices over time. **a** Fraction of varicella cases (%) generated by contacts of susceptible individuals of age i (x axis) with infected individuals of age j (y axis), as estimated by model M2 in the year 1992 (average over 100 simulation runs with the mean estimates of model parameters). **b** The same as **a** but for 2002. **c** The same as **a** but for 2012.

An increase in the proportion of infection transmission due to contacts among children aged 0–3 years from 1992 to 2012 is also detectable (Fig 3). Specifically, the estimated contribution of contacts among children 0–3 years to the infection transmission increased between 1992 and 2012 from 19.4% to 28.6%, while that of contacts among children 4–6 years decreased from 24.4% to 20.1%.

**Fig 4:**
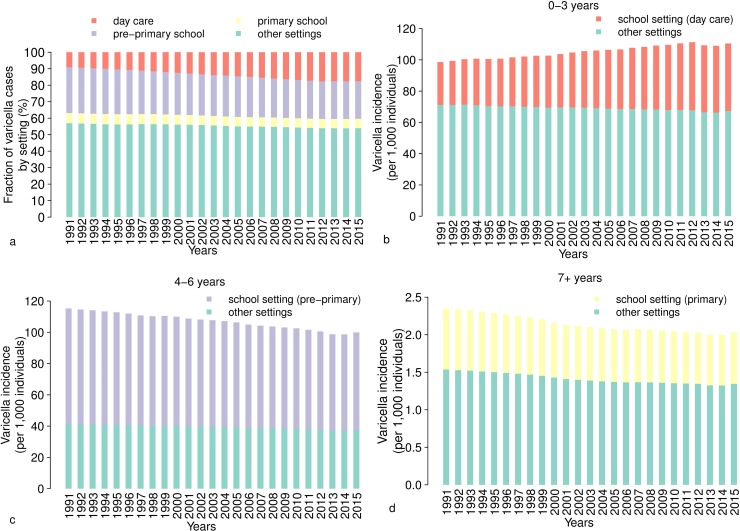
The contribution of schools to varicella transmission. **a** Estimates obtained with model M2 on temporal changes in the percentage of varicella cases generated in different settings (day care, pre-primary school, primary school and other settings) over the period 1991–2015. **b** Mean incidence of varicella in the age group 0–3 years (per 1,000 individuals) as estimated by model M2 over the period 1991–2015. Different colours represent the contribution of schools and other settings. **c** As **b** but for the age group 4–6 years. **d** As **b** but for the age group >7 years.

The increase of day-care contacts in the early childhood estimated by model M2 has an impact on the relative contribution of different settings to the overall transmission of varicella. According to our results, although the total incidence of varicella slightly decreased between 1991 and 2015 (from 12.1 to 11 cases per 1,000 individuals), the fraction of cases generated at school facilities of any level (i.e. day care, pre-primary and primary schools) raised from about 43.1% to 46.11% over the same period (see Fig 4A). Such increase is mainly driven by the changing role of day-care centers in varicella transmission, whose contribution to the total varicella cases rises from, on average, 9.1% in 1991 to 17.6% in 2015 (Fig 4A). In particular, infections among children under 3 years of age caused by day-care contacts raised from 27.8% in 1991 to 39.1% in 2015 (Fig 4B and S1 Text).

On the other hand, the percentage of the total yearly infections generated in pre-primary schools decreased from 27.8% to 22.8% and the fraction of infections in other settings diminished from 56.9% to 53.9% (Fig 4A). However, the contribution of different settings to infections occurring among individuals older than 4 years remained substantially unchanged (see Fig 4C and 4D).

## Discussion

Epidemiological data collected in France between 1991 and 2015 show that varicella incidence rates have remained approximately constant following a stabilization of the birth rate in the early 90’s [9,10]. This is coherent with our understanding of the role played by dynamics of the crude birth rate in shaping the transmission of childhood infections, such as measles and varicella [4–7,22]. Data disaggregated by age group however reveal that infection rates have increased in children younger than 3 years, therefore suggesting an increased circulation of varicella in early childhood. Similar patterns have been detected in other regions and countries, e.g. England, Slovenia and the US [11–16]. A plausible explanation for the observed epidemiological trends may rely on the increase of contacts among pre-school children, possibly caused by growing attendance rates at nurseries and day-care centers [11,12,23–25]. In this work, we investigated this hypothesis and showed that a progressive increase of mixing among 0–3 year-old children may have led to a 12% increase of varicella incidence in this age group between 1991 and 2015. Specifically, our results suggest that a 30% growth in the average number of day-care contacts may have increased by 50% the contribution of 0–3 year-old children to the overall number of yearly infections during the considered period. According to model estimates, although in the last decades the fraction of infections generated in schools of any level (day care included) remained rather stable around 45%, the fraction of transmission in day-care centers almost doubled during the same period.

From a policy-making perspective, our results suggest that the inclusion of children in day-care facilities or nurseries is expected to produce an earlier exposure to pathogens, increasing their risk of contracting infectious diseases. These findings may be useful to interpret results of policies (e.g. vaccination) and to design effective and targeted intervention strategies, e.g. scheduling age at vaccination. It is also important to note that a decrease in the age at infection for childhood diseases may be partially masked by rather stable incidence rates in the overall population.

The central role of age at entry in the community in the early childhood phase suggested by our analysis is widely supported by previous works. Silhol et al. [26] showed that the median age at varicella infection may be related to the fraction of children attending pre-schools, thus explaining the large variability in the age-specific seroprevalence observed across European countries [27]. Early exposure in day-care facilities was also suggested as a possible driver for the increase in varicella incidence reported in children from 12 months to 2 years in Slovenia in the period 1979–89 [12]. More generally, several studies identified a clear link between changes in mixing patterns due to the school calendar (e.g. school term vs. school holidays) and the strong seasonality of varicella dynamics [21,28–30]. A modeling study, based on theoretical age-specific contact patterns, suggested that the increase in GP varicella consultations rates observed in the UK between 1970 and 1998 among the youngest age segments of the population was compatible with an increase in early childhood contact rates [17].

Population surveys and modelling approaches have been proposed to investigate human mixing patterns by age, providing static estimates of country-specific contact matrices (e.g. [18,31–35]). The characterization of contacts was indeed found to be particularly important to achieve accurate and reliable modelling results and reduce uncertainties on recommendations for vaccination against varicella [36]. However, little is known to what extent mixing patterns by age may change over time, for instance, as a consequence of socio-demographic and legislative changes.

Our analysis represents a first step in this direction based on recent realistic estimates of age-specific contact rates in France and a rather simple assumption on how contacts in pre-school children may have changed as a consequence of increased day-care enrolment rates [24]. The model innovates on previous approaches also by explicitly taking into account the potential impact of demographic changes in shaping temporal changes in varicella circulation in the country [5–7].

The hypothesis of an increase in the number of contacts established by 0–3 years old infants at day-care facilities is supported by the increase in both the enrolment rates and the potential capacity of day-care services observed for this age group in France during the last decades [24,25].

Nonetheless, our study presents some limitations that call for a deeper understanding of temporal changes in mixing patterns to improve our interpretation of medium to long-term trends in the epidemiology of infectious diseases.

First, in our model we assume a linear increase in contacts rates of young children shaped by a unique scaling factor for all age groups. This assumption could be too simplistic to accurately reproduce the considered phenomenon. The inclusion of more flexible functional forms to describe temporal changes in contact rates would possibly improve model accuracy in reproducing varicella incidence over time for some age groups. Future modeling efforts in this direction would certainly benefit from cross-sectional and longitudinal studies showing how social mixing has changed over time.

The underestimation of varicella incidence in 3-year-old children with respect to reported data suggests that our model underestimated either the number of contacts or the transmission events in this age class. This may be partially due to mixed enrolment of children in different structures, as this age indicates the transition from day-care centers, potentially available for up to 54% of children (data for 2014 [25]), to pre-primary schools (ecoles maternelles), characterized by 100% attendance since 2000 [24]. Also, under the social contact hypothesis [20, 37] that is widely adopted for modeling childhood infections [38–40], here we consider age-specific transmission rates to be proportional to average daily contact rates through a single constant proportionality factor. However, it is possible that considering age-specific proportionality factors and taking into account the duration of social interactions may better describe the VZV transmission across different ages [35,41,42].

In our model, we did not consider the impact of changes of pre-school and school attendance on the school size. A study in the region of Corsica found that the age of varicella infection decreased as school size increased, likely due to an increased number of contacts per individual [43]. Including this aspect into our model is rather challenging because of the very diversified offer for early childhood services in France (different types of day-care centers, qualified nannies, qualified nannies at home, etc.) and the lack of a centralized management and registration [25].

Finally, additional mechanisms such as temporal changes in individual mixing outside schools, e.g. due to changes in household size and composition [44], may also play a role in the transmission dynamics of varicella. For example, a doubling varicella incidence was reported in Slovenia in the period 1979–1998 in 0 years old infants notwithstanding children less than 10 months are not accepted at day-care facilities [12]. Earlier varicella infections due to infected older siblings may explain the observed trends [43].

Our modelling results suggest that changes in mixing patterns at day-care structures represent one plausible component leading to the increase of incidence estimates over time in all corresponding age classes. The performed analysis focused on the epidemiology of varicella in France over the period 1991–2015. However, conclusions of this work may apply to other infections, such as measles and pertussis, and to countries that have undergone an increase in the school enrolment of young children, as is the case of England and Slovenia [11,12].

Future estimates on how the age-specific VZV immunity profiles have changed over time, based for instance on serological surveys conducted in different years, would help to exclude or quantify the contribution of other competing hypotheses to changes in day-care contact patterns in shaping the observed temporal variations in VZV incidence rates. For instance, while a constant reporting rate over time and across different ages was here assumed, changes in reporting behavior of individuals of different ages might have also occurred between 1991 and 2015.

Previous work, however, showed that extrapolated GP surveillance data were estimated to capture more than 96% of varicella cases in the 90’s in France [45], suggesting that no considerable improvement in consultation rate is thus possible.

## Supporting information

S1 Text Supporting text. Supporting text containing methodological details and additional results.
(PDF)
