# Supplementary material for: Modeling the impact of changes in day-care contact patterns on the dynamics of varicella transmission in France between 1991 and 2015
Source: PLoS Comput Biol. 2018 Aug 1;14(8):e1006334. doi: 10.1371/journal.pcbi.1006334 (PMC6089450; doi:10.1371/journal.pcbi.1006334)
Supplement: S1 Text — Supporting text containing methodological details and additional results. (PDF) [file pcbi.1006334.s001.pdf]

Modeling the impact of changes in day-care contact patterns on the dynamics of varicella transmission in France between 1991 and 2015

Valentina Marziano[1,#], Piero Poletti[1], Guillaume Béraud[2,3,4], Pierre-Yves Boëlle[5], Stefano Merler[1,⋆] and Vittoria Colizza [5,⋆]

[1]Center for Information Technology, Bruno Kessler Foundation, Trento, Italy
[2]Médecine Interne et Maladies Infectieuses, Centre Hospitalier de Poitiers, Poitiers, France
[3]EA2694, Université Droit et Santé Lille 2, Lille, France
[4]Interuniversity Institute for Biostatistics and statistical Bioinformatics, Hasselt University, Hasselt, Belgium
[5]INSERM, Sorbonne Université, Institut Pierre Louis d'Épidémiologie et de Santé Publique IPLESP, F75012 Paris, France

[#]Corresponding author
[⋆]These authors are joint senior authors on this work

Supporting Text

Contents

1 Materials and methods **2**
1.1 Epidemiological data . . . 2
1.2 The model . . . 4
1.3 Contact matrices over time . . . 5
1.4 Contribution of different settings and age segments to varicella transmission . . 7
1.5 Models' parametrisation . . . 7

2 Additional results **10**
2.1 Model M1 . . . 10
2.2 Model M2 . . . 12
2.3 Models' comparison . . . 13

1 Materials and methods

1.1 Epidemiological data

Varicella is monitored since 1991 by the French national system of clinical surveillance [1]. Incidence data and confidence intervals are estimated by the French GP surveillance system using a Poisson distribution to model the number of cases reported to GPs, and a normal-approximation for the computation of the 95% confidence intervals of incidence rates [2]. Estimates obtained from collected data revealed a stable incidence rate of varicella in the population during the period 1991 − 2015 (Figure S1). Although the total varicella incidence slightly decreased between 1991 and 2015 such variation is not statistically significant. In order to assess whether a temporal trend in incidence records is present we used a linear regression model to fit the observed yearly overall incidence of varicella over the period 1991 and 2015.
On the other hand, as shown in Figure S2, data disaggregated by age highlight a statistically significant increase of incidence rates in most of age groups below 4 years of age and a decrease of varicella cases among individuals older than 5 years.

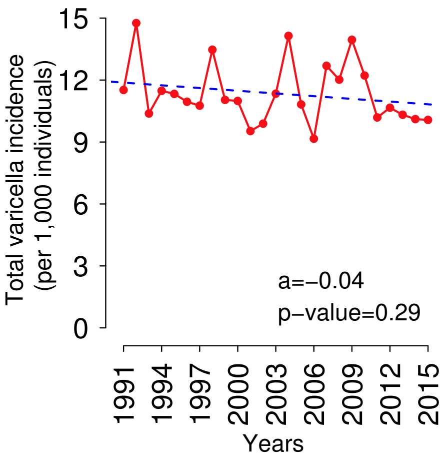

Figure S1: **Yearly total incidence of varicella based on the GPs Sentinelles Network (1991-2015).** Total incidence of varicella (per 1,000 individuals) as observed in France in 1991-2015 [1] (red) and as obtained by performing linear regression (blue). The p-value obtained indicates that there is no significant relationship between time and incidence level; a represents the slope of the linear model.

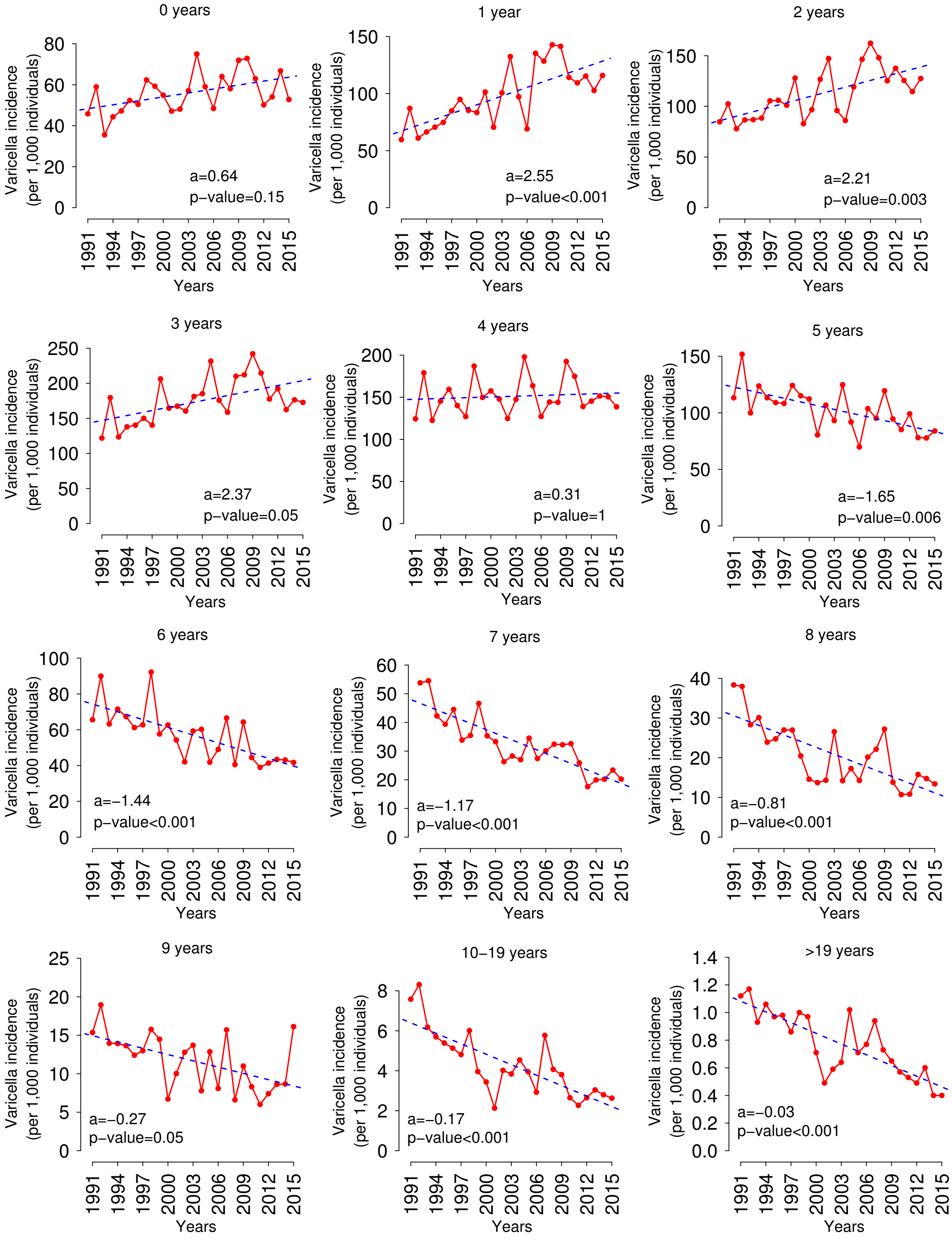

Figure S2: **Yearly incidence of varicella by age based on the GPs Sentinelles Network (1991-2015).** Incidence of varicella by age (per 1,000 individuals) as observed in France in 1991-2015 [1] (red) and as obtained by performing linear regression (blue). The slope of the linear model (a) is shown along with the corresponding p-value, Bonferroni corrected. Small p-values indicate that there is a significant relationship between time and incidence level.

1.2 The model

In this study we use a deterministic non-stationary age-structured model to simulate Varicella-Zoster Virus (VZV) transmission dynamics. In the model, the population is stratified into four epidemiological classes: individuals protected against varicella infection by maternal antibodies, individuals susceptible to varicella infection, individuals infected (who can transmit the infection) and individuals recovered from varicella. Recovered individuals are assumed to be life-long immune against VZV re-infection. Each epidemiological class is further divided in 90 one-year age groups (0-89+). Susceptible individuals are exposed to an age- and time-dependent force of infection of the following form:

$$\lambda_i(t) = \beta \sum_{j=0}^{n} C_{ij}(t) \frac{I_j(t)}{N_j(t)} \tag{1}$$

where β is a factor driving the contribution of individuals' contacts to the transmission of infection; $n = 89$ years; $C_{ij}(t)$ is the average number of contacts of an individual of age i with individuals of age j and $I_j(t)/N_j(t)$ is the fraction of infectious individuals of age j at time t. Following the approach proposed in previous studies [3, 4], the model explicitly accounts for the demographic dynamics of the population occurred between 1850 and 2015, mimicking time varying birth and age-specific mortality rates observed in France during the period considered [5] (see Figure S3). The system of ordinary differential equations describing epidemiological and demographic transitions within a given year is the following:

$$\begin{cases} \dot{M}_i(t) = \delta_{i0} b(t) N(t) - \omega M_i(t) - \mu_i(t) M_i(t) \\ \dot{S}_i(t) = \omega M_i(t) - \lambda_i(t) S_i(t) - \mu_i(t) S_i(t) \\ \dot{I}_i(t) = \lambda_i(t) S_i(t) - \gamma I_i(t) - \mu_i(t) I_i(t) \\ \dot{R}_i(t) = \gamma I_i(t) - \mu_i(t) R_i(t) \end{cases} \tag{2}$$

where

- $M_i(t)$ is the number of individuals of age i who are protected against varicella infection by maternal antibodies at time t;
- $S_i(t)$ is the number of susceptible individuals of age i at time t;
- $I_i(t)$ is the number of infected individuals of age i at time t;
- $R_i(t)$ is the number of recovered individuals of age i at time t;
- $N_i(t)$ is the number of individuals of age i at time t;
- $N(t) = \sum_{i=0}^{n} N_i(t)$ is the population size at time t; $n = 89$ years;
- $b(t)$ is the yearly crude birth rate observed in France at time t (Figure S3**a**);
- δ_{ij} is the Dirac delta function, i.e. $\delta_{ij} := \begin{cases} 1 & \text{if } i = j \\ 0 & \text{otherwise} \end{cases}$

- $\mu_i(t)$ is the yearly age-specific mortality rate observed in France (Figure S3**b**);
- $1/\omega = 2$ months is the average duration of protection conferred by the passive transfer of maternal antibodies, based on published estimates [6];
- γ is the recovery rate from varicella, set in such a way to reflect an average generation time of varicella equal to 3 weeks [7].

At the end of each year, the age of individuals aged 0-88 years is incremented by one.
Following the approach proposed in [3, 4] the demographic and transmission model was initialized according to the population age structure in 1850. The number of individuals in each of the considered epidemiological classes reflected the fraction of susceptible, infected and immune individuals associated with a certain parameter set and the corresponding equilibrium solution obtained by running the transmission model with constant fertility and mortality rates fixed to those observed in 1850 [5], and by initializing the system with 10 infected individuals in a fully susceptible population. Simulations of varicella dynamics from 1850 to 2015 were obtained by running the model with time varying crude birth and age-specific mortality rates, as observed in [5]. The demographic model is able to capture the changes in the age distribution of the French population during the whole considered period (see Figure S4).

After recovery of varicella, VZV may reactivate causing Herpes Zoster (HZ). The mechanism of VZV reactivation into HZ and the complex interplay between varicella and HZ disease has not yet been completely understood [8, 9]. Although zoster cases may transmit varicella [10], little is known about their relative infectiousness compared to varicella cases [9]. However, in settings where varicella routine vaccination has not yet been introduced, as is the case of France, zoster cases are much less frequent than varicella cases, thus, their contribution to VZV force of infection is likely to be negligible. For the above reasons, we decided to not include Herpes Zoster dynamics in our model.

1.3 Contact matrices over time

In this study we consider two variations of the general model described in Section 1.2, differing in the assumption made to model human mixing patterns over time.

- **Model M1.** The average number of contacts of an individual of age i with individuals of age j at time t is assumed to be

$$C_{ij}(t) = \bar{C}_{ij}^S + \bar{C}_{ij}^O$$

  where $\bar{C}_{ij}^S$ and $\bar{C}_{ij}^O$ are the average contact matrices estimated in 2012, respectively within and outside schools, for France [11]. In this model no changes in mixing patterns over time are assumed.

- **Model M2.** The average number of contacts of an individual of age i with individuals of age j is allowed to vary over time as a consequence of changes in the number of school contacts between 0-3 years old children as follows:

$$C_{ij}(t) = f_{ij}(t)\bar{C}_{ij}^S + \bar{C}_{ij}^O$$

where

$$f_{ij}(t) := \begin{cases} 1 - \alpha(2012 - t) & \text{if } i < 4 \text{ or } j < 4, \quad \alpha \in \mathbb{R} \\ 1 & \text{elsewhere} \end{cases} \tag{3}$$

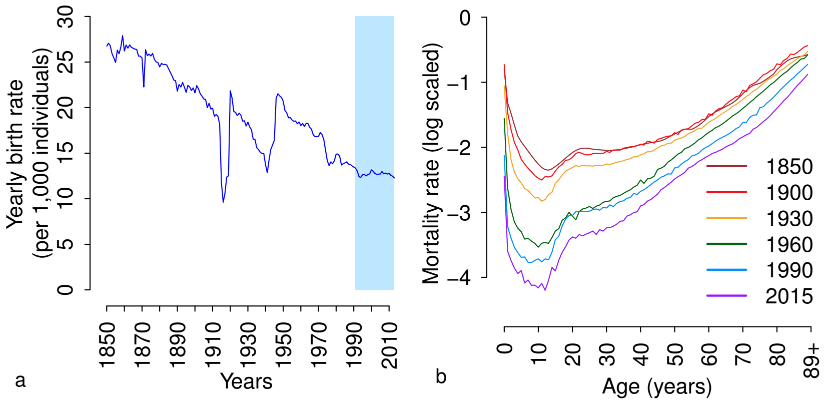

Figure S3: **Historical demographic data.** **a** Yearly birth rate as observed in France during the period 1850-2015 [5]. **b** Age-specific mortality rate as observed in France at different years [5].

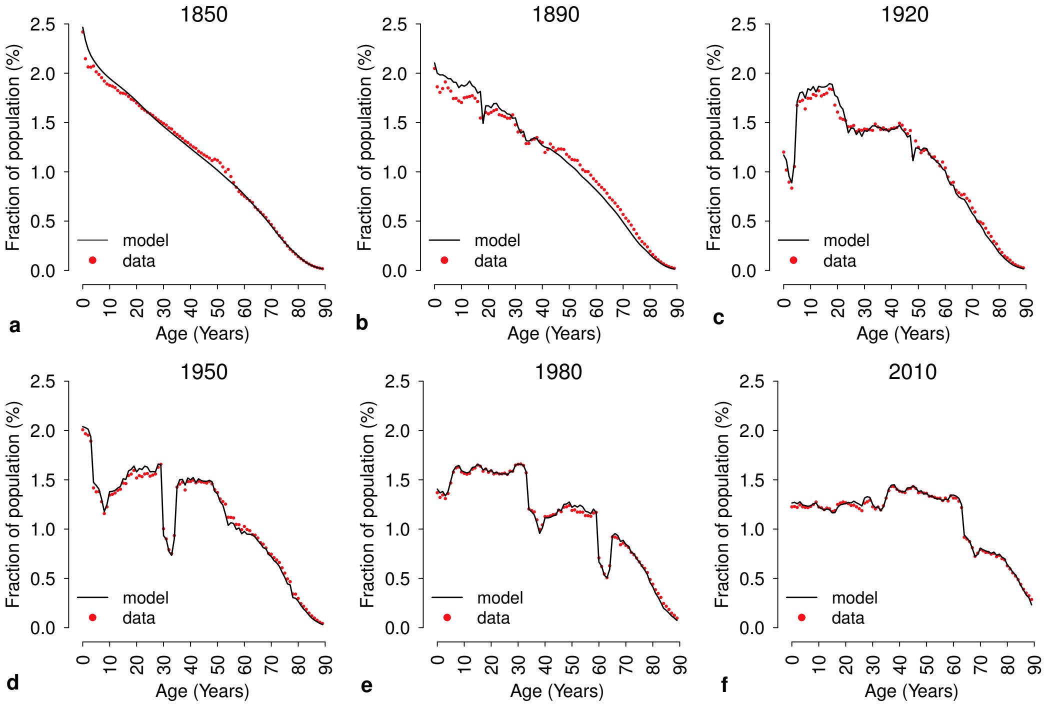

Figure S4: **Demographic validation of the model.** **a** Observed (red) and simulated (black) age distribution of the French population at different years of the simulated period (1850-2015).

1.4 Contribution of different settings and age segments to varicella transmission

The contribution of different settings and age segments to varicella transmission is computed by explicitly tracking the cumulative number of infections occurring at age i generated in a setting P by contacts with infected individuals of age j, through the following differential equation:

$$\dot{K}_{ij}^{P}(t) = \beta C_{ij}^{P}(t)\frac{I_j(t)}{N_j(t)}S_i(t)$$

where $C_{ij}^{P}(t)$ is the average number of contacts of an individual of age i with individuals of age j in the setting P at time t.
Specifically, the total number of cases of age i generated in a setting P by contacts with infected individuals of age j in the time interval $[t_1, t_2]$ is defined as:

$$K_{ij}^{P}(t_2) - K_{ij}^{P}(t_1)$$

For instance, the number of cases in individuals aged 4-6 years caused (in any setting) by contacts with individuals aged 0-3 years in $[t_1, t_2]$ was computed as:

$$\sum_{P\in\{S,O\}}\sum_{i=4}^{6}\sum_{j=0}^{3}[K_{ij}^{P}(t_2) - K_{ij}^{P}(t_1)]$$

Furthermore, we assume that infections caused by school contacts among individuals aged 0-3 years occur in day-care centers, infections caused by school contacts among individuals aged 4-6 years occur in pre-primary schools and infections caused by school contacts among individuals older than 7 years occur in primary schools or higher levels.

1.5 Models' parametrisation

The two models considered are parametrised separately by applying a Markov Chain Monte Carlo (MCMC) approach to the negative binomial likelihood of the yearly incidence of varicella by age observed in France between 1991 and 2015 [1].

Model M1 has the following free parameters:

- transmission scale factor β (see Equation 1);
- reporting rate of varicella, $\rho \in [0, 1]$, which is assumed to be constant over age and time;
- dispersion parameter of the negative binomial distribution x.

For each year y between 1991 and 2015 the negative binomial likelihood is computed as follows:

$$\mathcal{L}(c_k|\beta,\rho,x) = \prod_{y=1991}^{2015}\prod_{k\in K}\frac{\Gamma(x+c_k(y))}{\Gamma(x)(c_k(y))!}\left(\frac{\rho m_k(\beta,y)}{x+\rho m_k(\beta,y)}\right)^{c_k(y)}\left(\frac{x}{x+\rho m_k(\beta,y)}\right)^{x} \quad (4)$$

where

- K is the set of age groups in the varicella incidence observed in the year y [1], see Figure S2;
- $c_k(y)$ is the mean number of varicella cases in the k-th age group observed in year y [1];
- $m_k(\beta, y)$ is the yearly number of cases in the k-th age group estimated by model M1 in the year y.

Model M2 has an additional free parameter, shaping the change in school contacts of young children over time (α, in Equation 3). The likelihood function used to fit model M2 is the same used to fit model M1. For each model, we assumed uniform prior distributions on model parameters; specifically, the prior distribution of β, x and α were assumed uniform in [0,1000], while the one of ρ was assumed unifrom in [0,1]. We then determined the posterior distributions by using random-walk Metropolis-Hastings sampling based on normal jump [12]. The posterior distribution was computed by considering 30,000 simulations and a burn-in of 1,000 iterations. Figure S5-S6 show the trace plots associated with illustrative chains, as obtained through the MCMC approach for model M1 and M2 respectively.
It is worth noting that β represents a bifurcation parameter for the considered dynamical system so that changes in the frequency of epidemic oscillations occur over a certain critical threshold of this parameter. Specifically, for sufficiently high values of β, model M2 produces triennial instead of annual epidemics. As a consequence, the MCMC algorithm reject all values of β for which the estimated varicella incidence over time is not compatible (in timing and magnitude) with data (Figure S6**a**).
Epidemiological data used in this work are available at `http://sentiweb.fr` [1]. Contact matrices estimated for France can be found in [11].

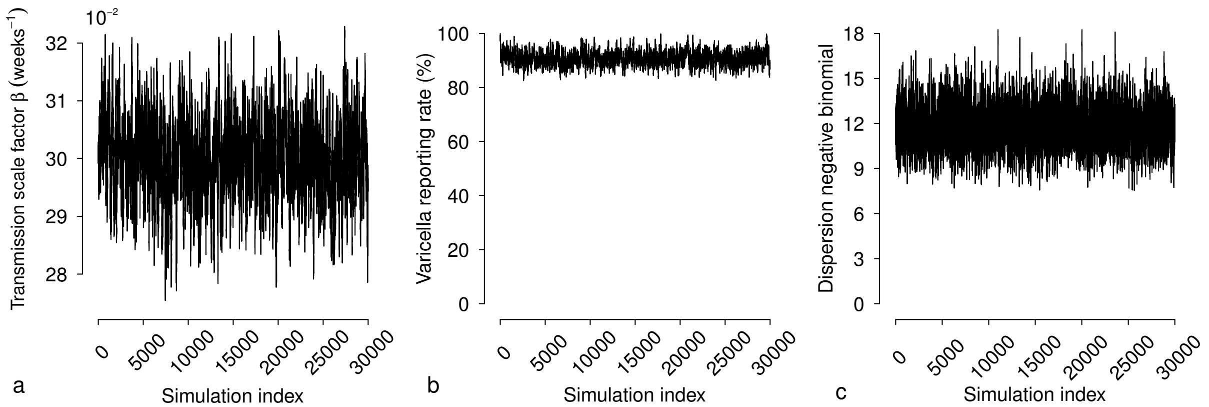

Figure S5: **Trace plots of MCMC chains (model M1).** **a** Transmission scale factor β (weeks^{-1}). **b** Varicella reporting rate ρ (%). **c** Dispersion parameter of the negative binomial distribution.

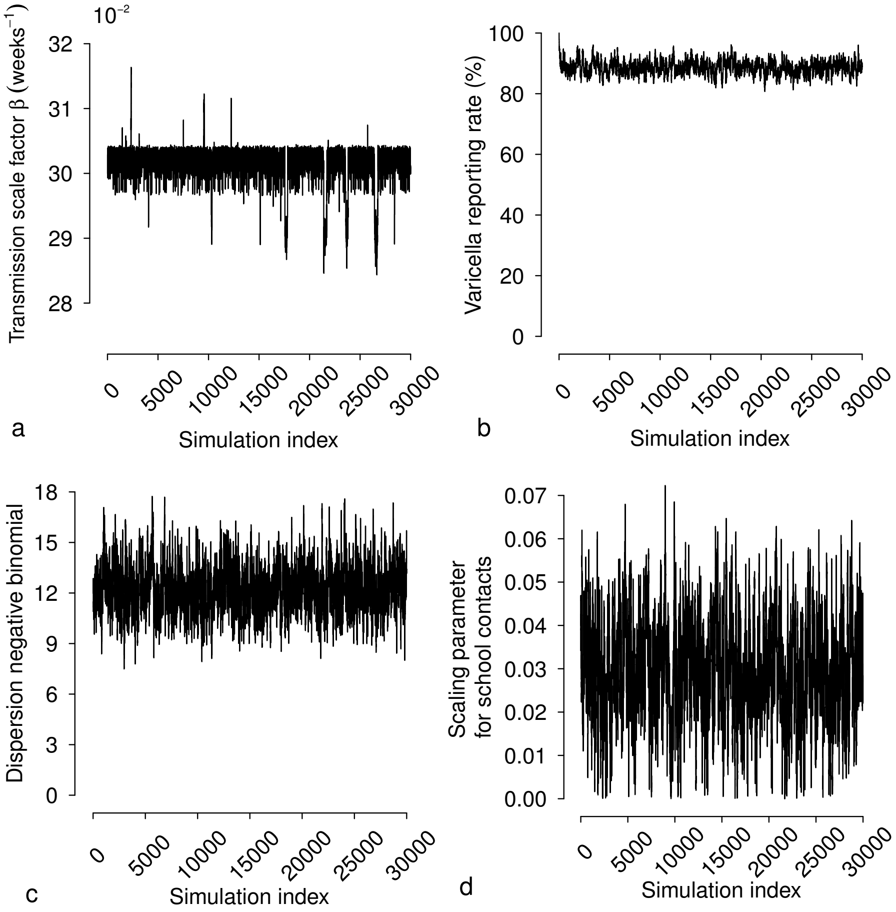

Figure S6: **Trace plots of MCMC chains (model M2).** **a** Transmission scale factor β (weeks^{-1}). **b** Varicella reporting rate ρ (%). **c** Dispersion parameter of the negative binomial distribution. **d** Parameter α (years^{-1}) shaping historical changes in school contacts of children aged 0-3 years.

2 Additional results

2.1 Model M1

According to model M1 the reduction observed in the French birth rate during the last century led to a decrease of varicella incidence in the population (see Figures S3 and S7**a**). Since the early 90's the birth rate remained approximately constant and the model estimates a roughly stable incidence, in good agreement with the observed data [1]. Model M1 is validated against the VZV serological profile observed in France in 2003 [13], which represents an independent dataset not used for model parameterisation. As shown in Figure S7**b**, the estimated seropositive fraction of the population at different ages lies in the 95% CI of the data for almost all age groups considered.

It is worth noting that model M1 estimates a stable varicella incidence in all age groups over the period 1991-2015 and thereby fails in capturing the temporal trends in the age-specific incidence of varicella observed in France (see Figure S8).

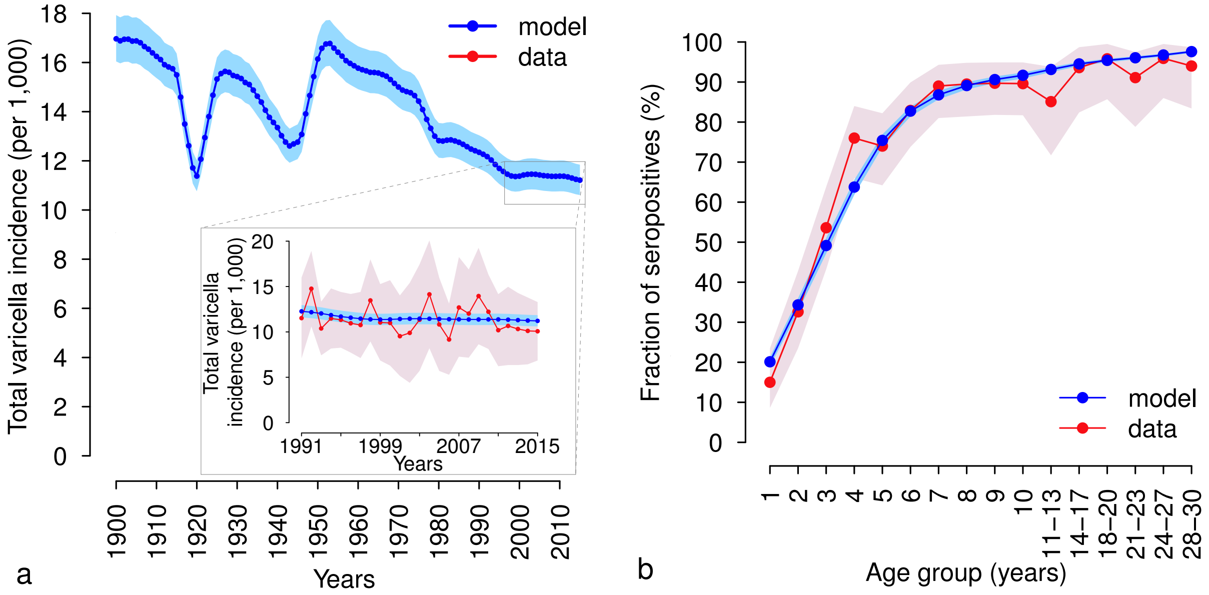

Figure S7: **Epidemiological validation of Model M1.** **a** Mean total incidence of varicella (per 1,000 individuals) as estimated by model M1 over the period 1900-2015. Shaded areas represent 95%CI of model estimates. The inset compares the total incidence of varicella predicted by the model in 1991-2015 (blue) to the one observed by the French GPs Sentinelles Network over the same period [1] (red). Shaded areas represent 95%CI of model estimates (light blue) and of the data (orange). **b** Age-specific VZV seroprevalence as observed in France in 2003 [13] (red) and as estimated by model M1 (blue). Shaded areas represent 95%CI of the data as computed by exact binomial test in [13] (orange) and 95%CI of model estimates (light blue).

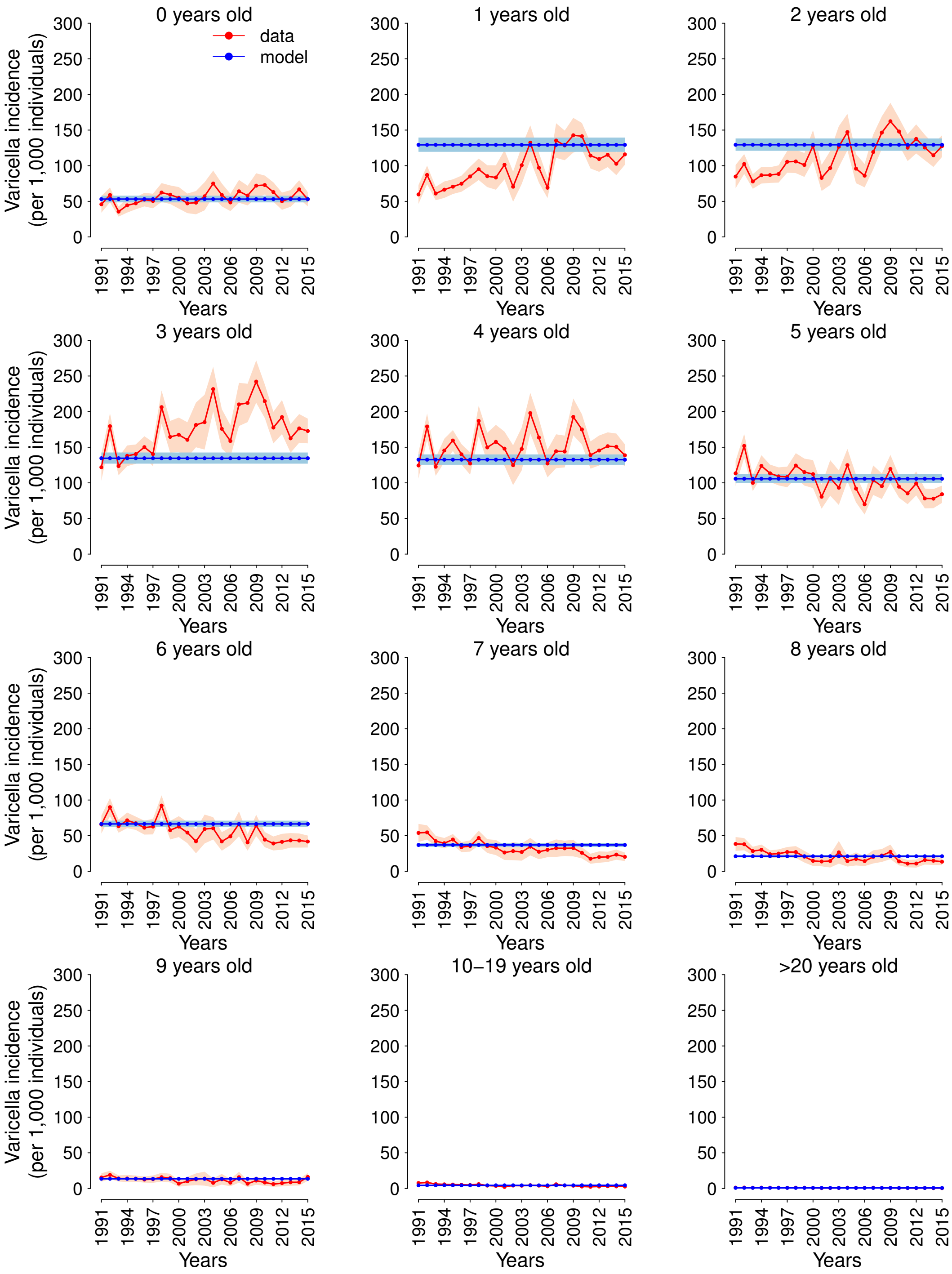

Figure S8: **Yearly incidence of varicella by age group (1991-2015).** Incidence of varicella by age as observed in France in 1991-2015 [1] (red) and as estimated by model M1 (blue). Shaded areas show the 95%CI of data and of model estimates.

2.2 Model M2

Figure S9 shows the temporal changes in mixing patterns as estimated by model M2 in terms of average contact matrices between individuals of different ages. According to the model, the number of the overall contacts among young children has progressively increased during the last decades. The observed changes are ascribable to an increase in the number of day-care contacts of children aged 0-3 years.

Simulations obtained with model M2 suggest that, although the overall incidence of varicella underwent a slight decrease between 1991 and 2015, the role of day-care centers in varicella transmission increased (see Figure S10**a**). Specifically, the increasing number of contacts at school among children aged 0-3 years resulted in an increase of the contribution of schools in the transmission of varicella in this age group (see Figure S10**b**). However, obtained estimates show that the relative weight of transmission in schools and other settings in older age groups did not significantly change over the considered period (Figure S10**c-d**).

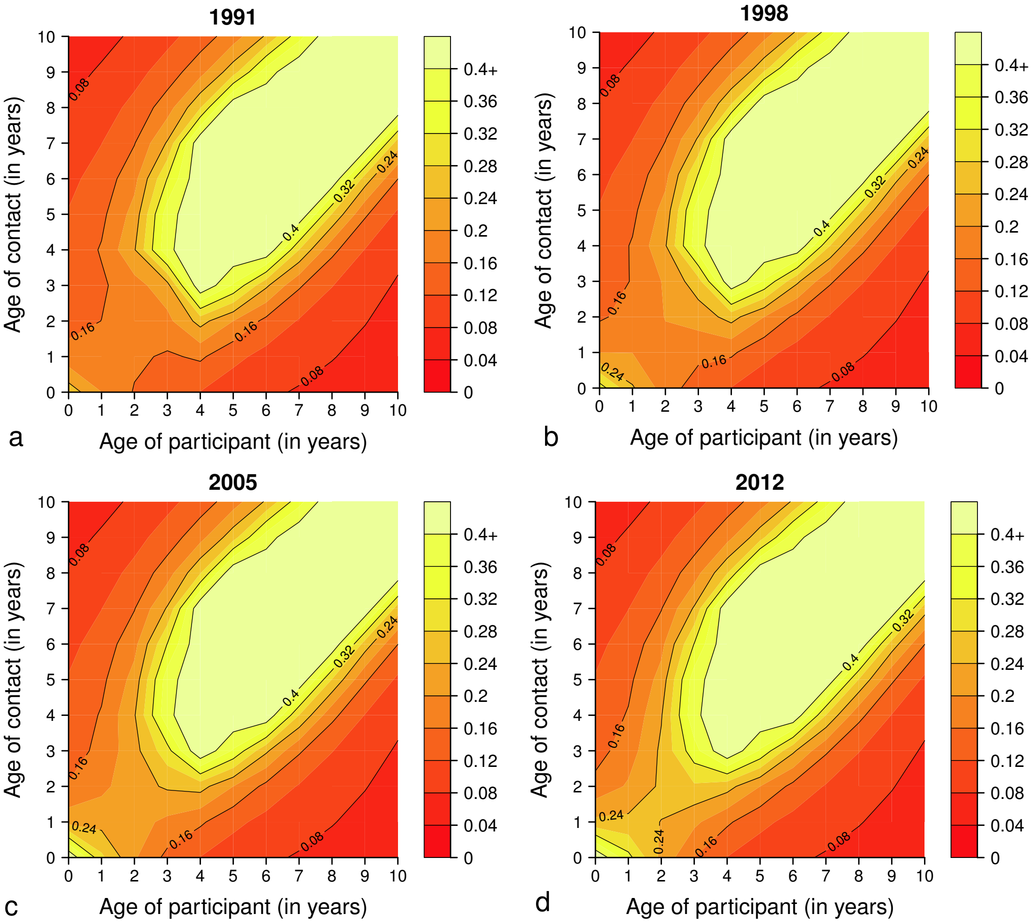

Figure S9: **Changes in mixing patterns between 1992 an 2012 (model M2).** Average contact matrices (total) for France by 1-year age-brackets, as obtained by model M2 between 1991 and 2012, when using the posterior mean estimate of α (see Equation 3).

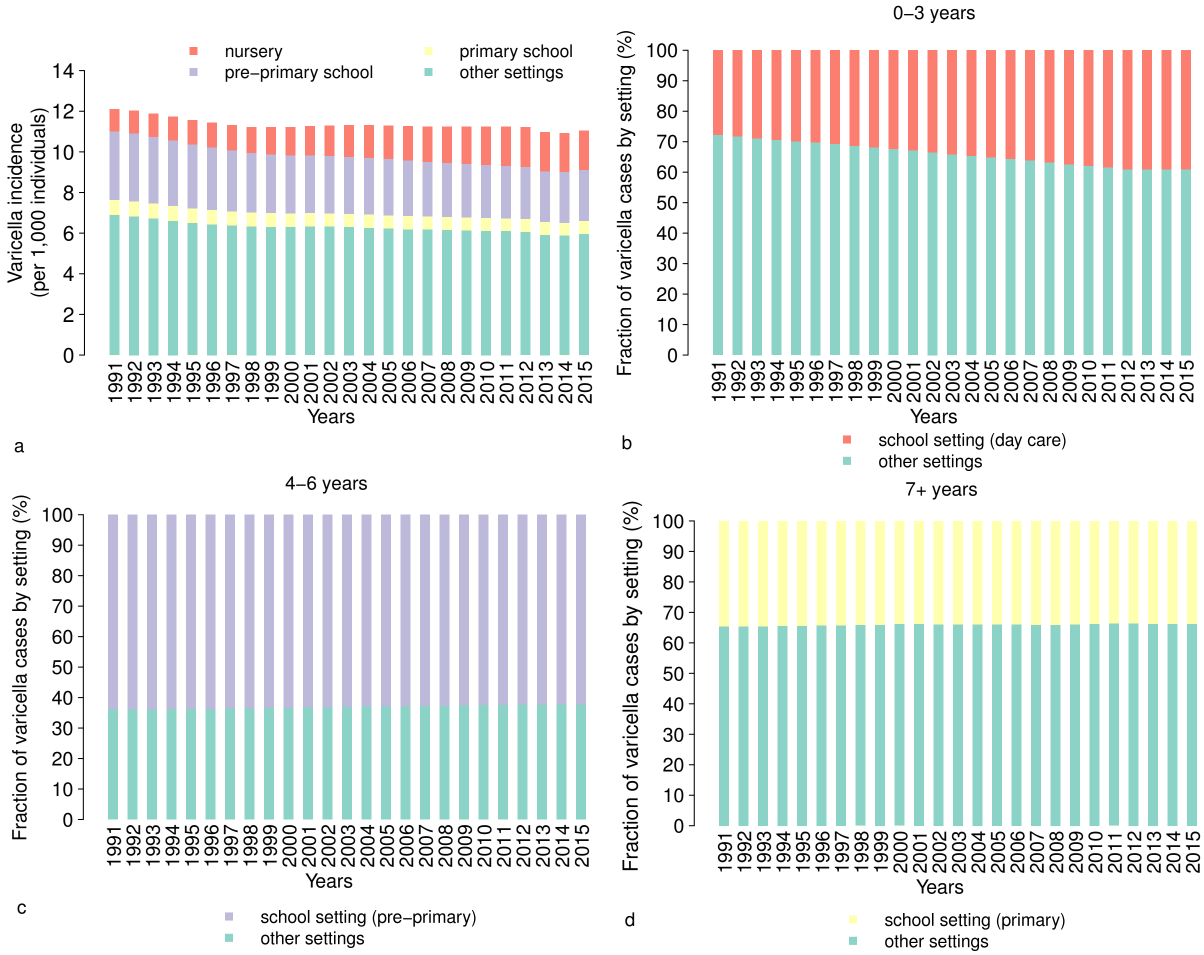

Figure S10: **Contribution of schools into varicella transmission.** **a** Estimates obtained with model M2 on temporal changes in the contribution of different settings to the mean total incidence of varicella (day care, pre-primary school, primary school and other settings) over the period 1991-2015. **b** Estimates obtained with model M2 on temporal changes in the percentage of varicella cases in the age group 0-3 years generated at school and in other settings. **c** As **b** but for the age group 4-6 years. **d** As **b** but for the age group > 7 years.

2.3 Models' comparison

Following the approach adopted in section 1.1, we performed - separately for each model and for different age groups - a linear regression analysis between time and the estimated incidence of varicella (see Table A). Obtained results show that model M2 better performs with respect to model M1 in reproducing age-specific temporal trends that were found significant in the data (see Table A and section 1.1).

Furthermore, possible Pearson's correlation was tested - separately for each age group and model - between the observed and estimated incidence (see Table B). While results obtained for model M2 show a positive and significant correlation in most of age groups, in the majority of cases the correlation associated with model M1 resulted either negative or not significant (see Table

B). These two simple analyses suggest that model M2 better represents the observed temporal patterns than model M1.

	Data		Model M1		Model M2	
Age group (years)	a	p-value	a	p-value	a	p-value
0	0.643	0.153	-0.001	< 0.001	0.577	< 0.001
1	2.553	< 0.001	-0.001	< 0.001	0.895	< 0.001
2	2.210	0.003	-0.001	0.014	0.560	< 0.001
3	2.372	0.051	-0.001	0.006	0.103	0.001
4	0.305	1.000	-0.001	0.009	-0.985	< 0.001
5	-1.649	0.006	0.000	0.159	-0.745	< 0.001
6	-1.443	< 0.001	0.000	0.222	-0.372	< 0.001
7	-1.165	< 0.001	0.000	1.000	-0.144	< 0.001
8	-0.810	< 0.001	0.000	1.000	-0.051	< 0.001
9	-0.272	0.047	0.000	1.000	-0.018	< 0.001
10-19	-0.175	< 0.001	0.001	1.000	0.002	0.809
20+	-0.026	< 0.001	-0.007	< 0.001	-0.006	< 0.001

Table A: Results obtained by performing for each age group a linear regression between incidence level and time as obtained when considering incidence as observed in the data (first two columns), as estimated with M1 (3rd and 4th columns) and as estimated with model M2 (5th and 6th columns). The slope of the linear model (a) is shown along with the corresponding p-value, Bonferroni corrected.

	Model M1		Model M2	
Age group (years)	r	p-value	r	p-value
0	-0.528	0.007	0.503	0.010
1	-0.570	0.003	0.759	< 0.001
2	-0.583	0.002	0.713	< 0.001
3	-0.413	0.040	0.689	< 0.001
4	-0.155	0.458	-0.106	0.614
5	0.213	0.308	0.649	< 0.001
6	0.116	0.581	0.723	< 0.001
7	0.237	0.255	0.846	< 0.001
8	0.026	0.903	0.704	< 0.001
9	0.074	0.726	0.584	0.002
10-19	-0.060	0.775	-0.259	0.210
20+	0.804	< 0.001	0.804	< 0.001

Table B: Results of Pearson's correlation (correlation coefficient r and p-value) between the observed and estimated incidence of varicella in different age groups for the two models.

References

[1] Réseau Sentinelles; INSERM, UPMC. http://www.sentiweb.fr.

[2] Turbelin C, Souty C, Pelat C, Hanslik T, Sarazin M, Blanchon T, et al. Age Distribution of Influenza Like Illness Cases during Post-Pandemic A(H3N2): Comparison with the Twelve Previous Seasons, in France. PLOS ONE. 2013 06;8(6):1–9. Available from: https://doi.org/10.1371/journal.pone.0065919.

[3] Merler S, Ajelli M. Deciphering the relative weights of demographic transition and vaccination in the decrease of measles incidence in Italy. Proceedings of the Royal Society of London B: Biological Sciences. 2014;281(1777). Available from: http://rspb.royalsocietypublishing.org/content/281/1777/20132676.

[4] Marziano V, Poletti P, Guzzetta G, Ajelli M, Manfredi P, Merler S. The impact of demographic changes on the epidemiology of herpes zoster: Spain as a case study. Proceedings of the Royal Society of London B: Biological Sciences. 2015;282(1804). Available from: http://rspb.royalsocietypublishing.org/content/282/1804/20142509.

[5] Human Mortality Database. University of California, Berkeley (USA) and Max Planck Institute for Demographic Research (Germany); [cited 19 April 2018]. Available from: http://www.mortality.org.

[6] Pinquier D, Gagneur A, Balu L, Brissaud O, Le Guen CG, Hau-Rainsard I, et al. Prevalence of anti-varicella-zoster virus antibodies in French infants under 15 months of age. Clinical and Vaccine Immunology. 2009;16(4):484–487.

[7] Metcalf CJE, Bjørnstad ON, Grenfell BT, Andreasen V. Seasonality and comparative dynamics of six childhood infections in pre-vaccination Copenhagen. Proceedings of the Royal Society of London B: Biological Sciences. 2009;276(1676):4111–4118.

[8] Guzzetta G, Poletti P, Del Fava E, Ajelli M, Scalia Tomba GP, Merler S, et al. Hope-Simpson's Progressive Immunity Hypothesis as a Possible Explanation for Herpes Zoster Incidence Data. American Journal of Epidemiology. 2013;177(10):1134–1142. Available from: http://dx.doi.org/10.1093/aje/kws370.

[9] Brisson M, Melkonyan G, Drolet M, Serres GD, Thibeault R, Wals PD. Modeling the impact of one- and two-dose varicella vaccination on the epidemiology of varicella and zoster. Vaccine. 2010;28(19):3385 – 3397. Available from: http://www.sciencedirect.com/science/article/pii/S0264410X10002604.

[10] Viner K, Perella D, Lopez A, Bialek S, Newbern C, Pierre R, et al. Transmission of Varicella Zoster Virus From Individuals With Herpes Zoster or Varicella in School and Day Care Settings. The Journal of Infectious Diseases. 2012;205(9):1336–1341. Available from: http://dx.doi.org/10.1093/infdis/jis207.

[11] Béraud G, Kazmercziak S, Beutels P, Levy-Bruhl D, Lenne X, Mielcarek N, et al. The French connection: the first large population-based contact survey in France relevant for the spread of infectious diseases. PloS one. 2015;10(7):e0133203.

[12] Gilks WR. Markov Chain Monte Carlo. Wiley Online Library; 2005.

[13] Khoshnood B, Debruyne M, Lançon F, Emery C, Fagnani F, Durand I, et al. Seroprevalence of varicella in the French population. The Pediatric infectious disease journal. 2006;25(1):41–44.
